# Flow cytometric immunobead assay for quantitative detection of platelet autoantibodies in immune thrombocytopenia patients

**DOI:** 10.1186/s12967-017-1317-2

**Published:** 2017-10-23

**Authors:** Juping Zhai, Mengyuan Ding, Tianjie Yang, Bin Zuo, Zhen Weng, Yunxiao Zhao, Jun He, Qingyu Wu, Changgeng Ruan, Yang He

**Affiliations:** 1 Ministry of Health Key Laboratory of Thrombosis and Hemostasis, Jiangsu Institute of Hematology, the First Affiliated Hospital of Soochow University, 188 Shizi St, Suzhou, 215006 Jiangsu China; 2 Cyrus Tang Hematology Center and Ministry of Education Engineering Center of Hematological Disease, and the Collaborative Innovation Center of Hematology, Soochow University, Suzhou, 215006 China

**Keywords:** Immunobead assay, Immune thrombocytopenia, Platelet glycoprotein, Quantitative flow cytometry, Treatment monitoring

## Abstract

**Background:**

Platelet autoantibody detection is critical for immune thrombocytopenia (ITP) diagnosis and prognosis. Therefore, we aimed to establish a quantitative flow cytometric immunobead assay (FCIA) for ITP platelet autoantibodies evaluation.

**Methods:**

Capture microbeads coupled with anti-GPIX, -GPIb, -GPIIb, -GPIIIa and P-selectin antibodies were used to bind the platelet-bound autoantibodies complex generated from plasma samples of 250 ITP patients, 163 non-ITP patients and 243 healthy controls, a fluorescein isothiocyanate (FITC)-conjugated secondary antibody was the detector reagent and mean fluorescence intensity (MFI) signals were recorded by flow cytometry. Intra- and inter-assay variations of the quantitative FCIA assay were assessed. Comparisons of the specificity, sensitivity and accuracy between quantitative and qualitative FCIA or monoclonal antibody immobilization of platelet antigen (MAIPA) assay were performed. Finally, treatment process was monitored by our quantitative FCIA in 8 newly diagnosed ITPs.

**Results:**

The coefficient of variations (CV) of the quantitative FCIA assay were respectively 9.4, 3.8, 5.4, 5.1 and 5.8% for anti-GPIX, -GPIb, -GPIIIa, -GPIIb and -P-selectin autoantibodies. Elevated levels of autoantibodies against platelet glycoproteins GPIX, GPIb, GPIIIa, GPIIb and P-selectin were detected by our quantitative FCIA in ITP patients compared to non-ITP patients or healthy controls. The sensitivity, specificity and accuracy of our quantitative assay were respectively 73.13, 81.98 and 78.65% when combining all 5 autoantibodies, while the sensitivity, specificity and accuracy of MAIPA assay were respectively 41.46, 90.41 and 72.81%.

**Conclusions:**

A quantitative FCIA assay was established. Reduced levels of platelet autoantibodies could be confirmed by our quantitative FCIA in ITP patients after corticosteroid treatment. Our quantitative assay is not only good for ITP diagnosis but also for ITP treatment monitoring.

**Electronic supplementary material:**

The online version of this article (doi:10.1186/s12967-017-1317-2) contains supplementary material, which is available to authorized users.

## Background

Immune thrombocytopenia (ITP) is an autoimmune disease characterized by reduced platelet counts (< 100 × 10^9^/L) and an increased risk of bleeding [1]. The pathophysiology underlying ITP is not completely understood. To date, several disease mechanisms have been proposed, including autoantibodies and cytotoxic T cells that target platelets and/or megakaryocytes. It has been shown that autoantibodies targeting surface glycoproteins on platelets and/or megakaryocytes account for more than half of ITP patients [2–4], and altering platelet clearance or inhibiting platelet production could be exerted by different types of antibodies in individual ITP patient [5]. The importance of platelet autoantibodies is self-evident in these ITP patients. Most recently, it was reported that the type and persistence of platelet autoantibodies are associated with refractoriness to rituximab or disease severity [6–8]. Based on these findings, detection of presence and quantification of platelet autoantibodies may be helpful in ITP prognosis or personalized treatment.

Currently, several methods have been developed for platelet autoantibody detection, including enzyme-linked immunosorbent assay (ELISA)-based monoclonal antibody immobilization of platelet antigen (MAIPA) assay and immunobead-based radioimmune assay (RIA) [9–12]. However, procedures in these assays are cumbersome and time-consuming for multiple antibodies detection, which hampers the wide use of these assays in most hospital laboratories [13]. Flow cytometric immunobead array (FCIA) is a recently developed technique, in which flow cytometry can detect multiple kinds of antibody-coated polystyrene microbeads that bind to several specific antigens  [14, 15]. Compared with previous MAIPA- and RIA-based assays, this FCIA assay is more rapid and simpler for multiple antibodies detection, which can be used in hospital settings to improve ITP diagnosis [16, 17]. However, lacking of quantitative property of this assay could result in high variations and failure to compare data from different sources, such as various disease courses or before and after treatment.

In this study, we developed a novel quantitative FCIA assay, which is suitable for quantitative measurement of platelet autoantibodies in ITP patients. Our results showed that this new quantitative FCIA assay can be used for ITP diagnosis. More importantly, our assay allows monitoring platelet autoantibody level changes in ITP patients before and after corticosteroid treatment, providing important information regarding patient responses and disease prognosis.

## Methods

### Study design and subjects

This was a case–control study. A total of 250 consecutive ITP patients, 163 non-ITP patients and 243 healthy controls from the First Affiliated Hospital of Soochow University between June 2012 and October 2016 were included in this study. ITP diagnosis was based on the guideline of American Society of Hematology [18]. All the cases were divided into 3 sets. Test set 1 was used to evaluate quantitative FCIA assay and its comparison with qualitative FCIA assay, including 201 ITP patients and 126 non-ITP patients presented with thrombocytopenia (including 66 leukemia; 20 aplastic anemia; 10 lymphoma; 9 myelodysplasia syndrome; 9 solid tumors after chemotherapy; 9 liver cirrhosis and 3 multiple myeloma), and 207 healthy individuals. Based on disease duration, the ITP patients were further classified into three subgroups, i.e. newly-diagnosed (< 3 months of diagnosis), persistent (3–12 months) and chronic (duration of ≥ 12 months) [19]. Test set 2 was used for comparison of platelet autoantibodies detection between quantitative FCIA and MAIPA assay, including 41 ITP patients, 37 non-ITP patients and 36 healthy controls. A pilot set was used to evaluate the change of platelet autoantibody levels before and after treatment, including 8 newly-diagnosed ITP patients (n = 8; 3 males and 5 females). Among them, 7 were treated with corticosteroids and one with corticosteroids and azathioprine. Levels of plasma platelet autoantibodies were measured before and 1 month after the treatment. Blood platelet counts in ITP and non-ITP patients were < 100 × 10^9^/L, whereas platelet counts in healthy controls were > 100 × 10^9^/L. The characteristics of all included subjects were shown in Table 1. All the included patients did not receive any medical treatment for at least 1 month before sampling.

**Table 1:** Subjects characteristics

Characteristics	Test set 1					Test set 2			Pilot set
	ITP (n = 201)			Non-ITP (n = 126)	HC (n = 207)	ITP (n = 41)	Non-ITP (n = 37)	HC (n = 36)	ITP (n = 8)
Disease subtype (n)	Newly-diagnosed (63)	Persistent (60)	Chronic (78)	NA	NA	NA	NA	NA	Newly-diagnosed (8)
Gender (female/male, n)	45/18	38/22	48/30	56/70	76/131	24/17	11/26	13/23	5/3
Age (median, range, years)	53 (18, 86)	53 (9, 86)	54 (13, 83)	44 (13, 88)	48 (24, 91)	33 (5, 68)	42 (9, 64)	30 (21, 50)	38.5 (9, 69)
Platelet count (× 10^9^/L)^a^	29.0 ± 16.9	41.0 ± 20.0	42.4 ± 20.3	41.5 ± 21.8	216.1 ± 56.8	38.2 ± 32.3	42.2 ± 31.0	211.4 ± 32.4	17.8 ± 9.9
Treatment (n, %)^b^	NA	58 (96.7)	78 (100)	NA	NA	27 (65.9)	NA	NA	NA
Steroids	NA	51 (85.0)	75 (96.2)	NA	NA	27 (65.9)	NA	NA	NA
Immunosuppressants	NA	15 (25.0)	27 (34.6)	NA	NA	10 (24.4)	NA	NA	NA
Splenectomy	NA	0 (0)	3 (3.8)	NA	NA	0 (0)	NA	NA	NA
Others	NA	10 (16.7)	23 (29.5)	NA	NA	5 (12.2)	NA	NA	NA

Data are presented as mean ± SD, or percentage in parenthesis

Steroids included dexathemethasone, prednisone or methylprednisolone; immunosuppressants included Cyclosporine A (CSA), Azathioprine (Aza) or Vindesine (VDS); others included Intravenous Immunoglobulin (IVIg), thrombopoietin (TPO) and Rituximab

*HC* healthy controls, *NA* not applicable or not available

^a ^Platelet count at first visit

^b^All the included patients did not receive any medical treatment for at least 1 month before sampling

### Sample preparation

Peripheral venous blood samples were collected into tubes containing EDTA as an anticoagulant. Plasma was separated by centrifugation, 3000 rpm for 10 min at room temperature, and stored at − 80 °C until further use. Normal platelets from healthy volunteers were isolated from peripheral blood and washed as described previously [17]. Plasma (100 µL) from patients or control subjects was incubated with 100 μL of normal washed platelets (1 × 10^9^/mL) at room temperature for 1 h. This allowed autoantibodies in plasma to bind to antigens on normal platelets. After 3 washes with phosphate-buffered saline (PBS) containing 0.05% EDTA, platelets were lysed in PBS containing 1% Triton X-100. The platelet lysate was centrifuged at 3000*g* for 20 min at 4 °C. The supernatant containing platelet protein and autoantibody complexes was used for assay analysis.

### Antibodies and microbeads

A goat anti-human IgG polyclonal antibody and human-IgG were purchased from Solarbio (Beijing, China). Monoclonal antibodies against human platelet glycoproteins GPIX (SZ1), GPIb (SZ2), GPIIIa (SZ21), GPIIb (SZ22) and P-selectin (SZ51) were generated in our laboratory, as described previously [11, 20–24]. A fluorescein isothiocyanate (FITC)-conjugated goat anti-human IgG (FITC-GAH) and a goat anti-mouse IgG (FITC-GAM) antibodies were purchased from Beckman Coulter (CA, USA). A FITC-labeled rabbit anti-goat IgG (FITC-RAG) was from Abcam (Cambridge, UK). Polystyrene microbeads (4 μm in diameter) with 8 different fluorescent intensities were obtained from Spherotech (Libertyville, IL, USA).

### Antibody coupling to microbeads

In this study, two sets of microbeads were prepared: one set with 6 different fluorescent intensities coupled to GAH for quantitative standard curves; another set with 5 different fluorescent intensities coupled to the monoclonal antibodies against human platelet glycoproteins GPIX (SZ1), GPIb (SZ2), GPIIIa (SZ21), GPIIb (SZ22) and P-selectin (SZ51) for autoantibodies detection. The coupling of the antibodies to the microbeads was carried out according to the manufacturer’s instructions. Briefly, a standard checkerboard titration method was used to determine the optimal antibody concentration for microbeads coupling. Then, microbeads were incubated overnight with the GAH antibody (30 μg/mL) or the monoclonal antibodies against platelet glycoproteins (50 μg/mL) at 4 °C in a sodium acetate buffer (0.1 M; pH 6.0), followed by 2 h incubation at room temperature with 2% bovine serum albumin (BSA) to block non-specific binding sites. The antibody-coupled microbeads were washed 3 times with a 0.05% Tween-PBS buffer and stored at 4 °C in a PBS buffer containing 0.02% sodium azide and 0.05% BSA. The stability of stored antibody-coupled microbeads was assessed over a 5-month time period.

### Quantitative FCIA

GAH-coupled microbeads with 6 different fluorescent intensities were incubated separately with 6 different concentrations of human IgG (20, 80, 160, 320, 640 and 1280 ng/mL) on a gentle shaker at room temperature. After 1 h, the microbeads were washed with a 0.05% Tween-PBS buffer and incubated with a FITC-GAH antibody. The microbeads were used for mean fluorescent intensity (MFI) evaluation by flow cytometry (CyAn™ ADP Analyzer, Beckman-Coulter, CA, USA). A standard curve was calculated based on the MFI of each type of GAH-coupled microbeads and corresponding concentrations of human IgG (Fig. 2a, b).

**Fig. 1:**
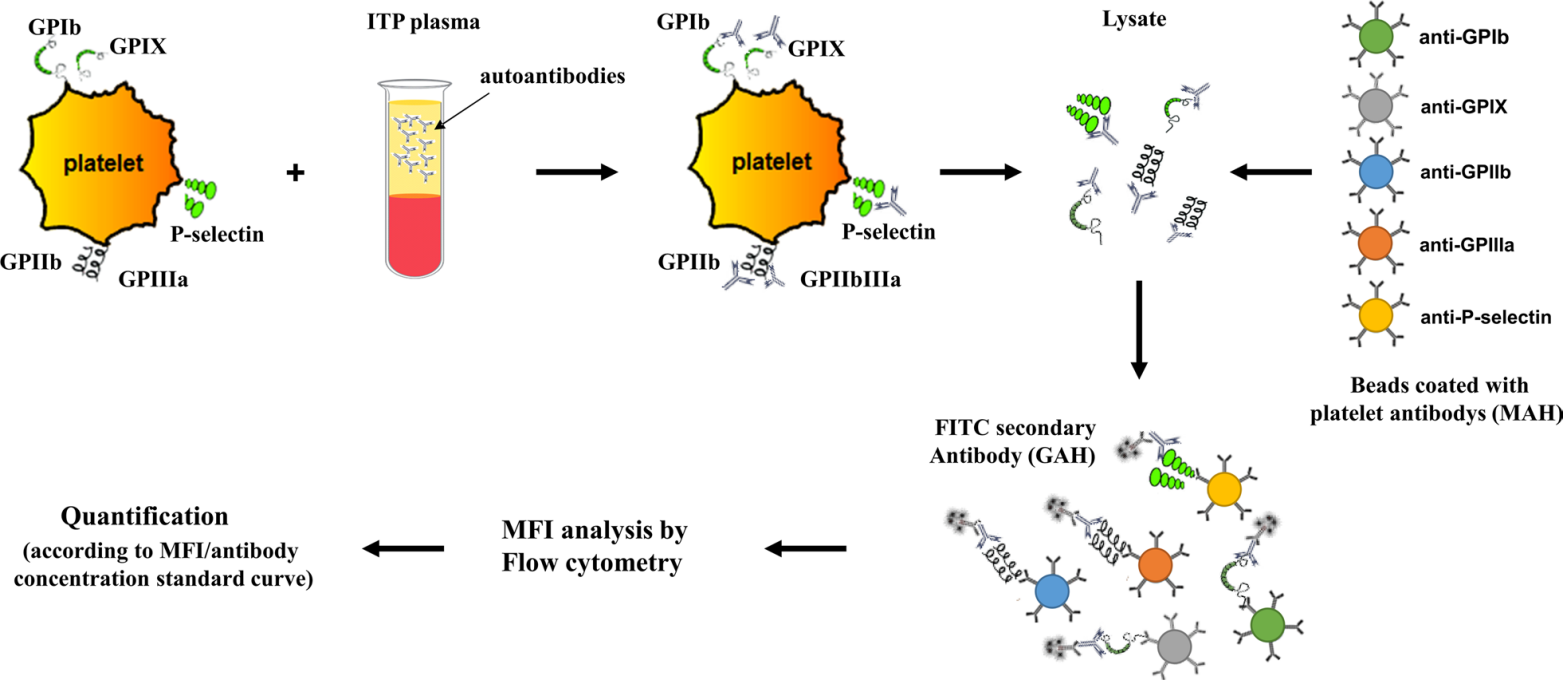
The principle and major steps of the FCIA assay. Platelet autoantibodies in ITP patient plasma were incubated with isolated normal platelets. The platelets were washed and lysed. Autoantibody-platelet antigen complexes were captured by microbeads coated with anti-platelet glycoprotein monoclonal antibodies (in different colors). The autoantibodies bound to microbeads were analyzed by flow cytometry using a FITC-conjugated goat anti-human IgG antibody. Levels of autoantibodies were determined based on values of mean fluorescence intensity (MFI) and standard corves with exogenous human IgG

Quantitative FCIA was carried out in parallel with the GAH for the standard curve and monoclonal antibodies against platelet glycoproteins GPIX (SZ1), GPIb (SZ2), GPIIb (SZ22), GPIIIa (SZ21) and P-selectin (SZ51) for platelet autoantibody detection. Fifty microliters microbeads coupled to antibodies SZ1, SZ2, SZ21, SZ22 and SZ51 were incubated with platelet lysates containing platelet antigen-autoantibody complexes at room temperature for 1 h with gentle shaking. The microbeads were washed with PBS by centrifugation at 4 °C, re-suspended in 0.5 mL PBS and analyzed for MFI by flow cytometry as described above (Fig. 1). Levels of each autoantibody were calculated based on the standard curve.

To evaluate assay variation, intra-assay coefficients of variation (CVs) were determined by 20 independent measurements with same sets of lysate samples in 1 day. Inter-assay CVs were assessed by measurements with same sets of lysate samples on 20 different days. Serial dilutions of plasma samples from ITP patients and normal individuals were used to assess assay sensitivity.

### Qualitative FCIA

The qualitative FCIA was performed as described previously [17]. The results were compared with those from the quantitative FCIA established in this study. For autoantibody detection, MFI values were obtained for each sample. A positive result was defined as the MFI value greater than the mean MFI value + 2 standard deviations (SD) from healthy controls.

### MAIPA assay

The MAIPA assay was performed in parallel to detect platelet autoantibodies using plasma from test set 2. The assay was done as described previously [9]. In short, the platelets for healthy donors were incubated with patient plasma and washed 3 times with PBS containing 0.05% EDTA. After that the platelets were solubilized with PBS containing 1% Triton X-100. The soluble platelet lysate (100 μL) was added to 96-well plates pre-coated with individual monoclonal antibodies SZ1, SZ2, SZ21, SZ22 or SZ51. After 1 h incubation and washing by PBS containing 0.05% Tween-20, horseradish peroxidase (HRP)-conjugated GAH antibody and HRP substrate 3,3′,5,5′-tetramethylbenzidine (TMB) was added to visualize the reaction.

### Statistical analysis

Statistical analysis was performed using SPSS v20.0 (Chicago, IL, USA) and and GraphPad Prism v5.0 (San Diego, CA, USA) softwares. Data are presented as mean ± SD. CV value was defined as the ratio of the SD to the mean. In reproducibility studies, samples from 30 randomly selected ITP patients were used to compare CV values from quantitative and qualitative FCIA methods. Student *t* test and one way ANOVA test were used for two- and multiple-group comparisons, respectively. Chi square test was used to compare the sensitivity, specificity, and accuracy between quantitative and qualitative assays for ITP diagnosis. Receiver operating characteristic (ROC) curves were plotted and areas under the curves (AUC) with 95% confidence interval (CI) were calculated to evaluate the efficacy of quantitative FCIA for ITP diagnosis. p < 0.05 was considered as statistically significant.

## Results

### Stability of antibody-coupled microbeads

To evaluate the potential long-term use of the antibody-coupled microbeads, we tested the stability of the antibody-coupled microbeads. MFI signals for microbeads-coupled to GAH with 6 different fluorescent intensities were recorded by flow cytometry. The values were 232.1 ± 4.8, 243.3 ± 0.8, 215.6 ± 3.9, 237.4 ± 5.6, 237.4 ± 5.6, 237.1 ± 1.9, respectively. We also assessed the MFI signals from microbeads coupled to the monoclonal antibodies against human platelet glycoproteins GPIX (SZ1), GPIb (SZ2), GPIIb (SZ22), GPIIIa (SZ21) and P-selectin (SZ51). The values were 758.6 ± 7.4 (SZ1), 732.1 ± 7.5 (SZ2), 555.3 ± 4.9 (SZ21), 747.3 ± 9.1 (SZ22), and 541.4 ± 6.7 (SZ51), respectively. The experiments were repeated 150 days later. The calculated CV values were 3.9% (GAH), 2.2% (SZ1), 2.1% (SZ2), 2.9% (SZ21), 2.2% (SZ22), and 2.7% (SZ51), respectively (See Additional file 1: Figure S1), indicating that the antibody-coupled microbeads were stable under our experimental conditions.

### Quantitative FCIA assay evaluation

**Fig. 2:**
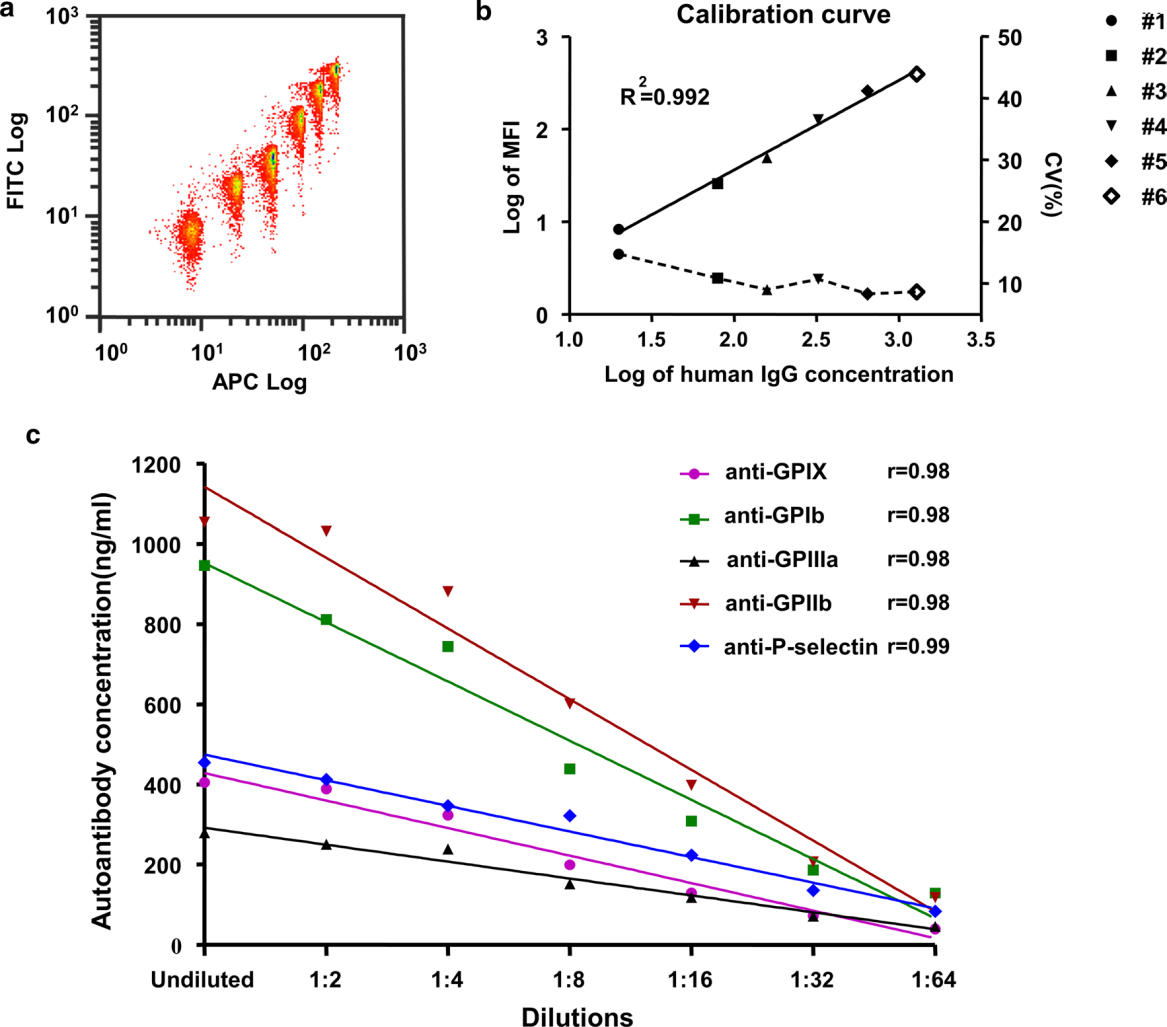
Detection of platelet autoantibodies with the quantitative FCIA assay. **a** Representative scatter plots of 6 types of microbeads (APC fluorescence) with increasing concentrations of antibodies (FITC fluorescence). **b** Calibration curve (solid line) and precision profiles (dashed line) of quantitative FCIA assay. A merged fitting curve was calculated by using the Log MFI values of six different types of microbeads with different fluorescence intensities (#1–6) and increasing concentrations of exogenous human IgG. CV: coefficient of variations. **c** Quantification of autoantibodies against different platelet glycoproteins by the FCIA. Diluted plasma samples from ITP patients containing autoantibodies against platelet GPIX (●), GPIb (■), GPIIIa (▲), GPIIb (▼) and P-selectin (◆) were evaluated by the quantitative FCIA. Autoantibody concentrations were calculated and curves were fitted. Significant correlations were found between diluted plasma samples and the autoantibody concentrations

We analyzed intra- and inter-assay variations of the quantitative FCIA using plasma samples from ITP patients. A representative scatter plot of six microbeads (APC fluorescence) with a series of concentration of human IgG (FITC fluorescence) was shown) (Fig. 2a). Based on the standard curve (Fig. 2b), intra- and inter-assay CVs were calculated. The intra-assay CVs and corresponding levels of autoantibodies against platelet GPIX, GPIb, GPIIIa, GPIIb and P-selectin were 2.9% and 336.1 ± 9.7 ng/mL, 3.1% and 682.3 ± 20.8 ng/mL, 4.2% and 227.4 ± 9.5 ng/mL, 5.9% and 904.1 ± 53.1 ng/mL, 6.0% and 365.3 ± 22.0 ng/mL, respectively. The inter-assay CVs and corresponding levels of autoantibodies against platelet GPIX, GPIb, GPIIIa, GPIIb and P-selectin were 10.5% and 346.5 ± 36.3 ng/mL, 7.3% and 687.8 ± 50.2 ng/mL, 9.9% and 314.1 ± 31.1 ng/mL, 6.7% and 1062.8 ± 70.9 ng/mL, 10.3% and 396.9 ± 40.9 ng/mL, respectively.

We also compared the reproducibility of the quantitative and qualitative FCIA assays (Table 2). The CV values of the quantitative FCIA assay for anti-GPIX, GPIb, GPIIIa, GPIIb and P-selectin autoantibodies were 9.4, 3.8, 5.4, 5.1 and 5.8%, respectively, whereas the CV values of qualitative FCIA assay for anti-GPIX, GPIb, GPIIIa, GPIIb and P-selectin autoantibodies were 28.9, 46.8, 36.3, 40.8 and 38.4%, respectively. The results indicate that the quantitative FCIA assay has a better reproducibility under our experimental conditions.

**Table 2:** Reproducibility of quantitative FCIA assay when compared to qualitative FCIA

Times of repetition	Anti-GPIX (%, n/n)		Anti-GPIb (%, n/n)		Anti-GPIIIa (%, n/n)		Anti-GPIIb (%, n/n)		Anti-P-selectin (%, n/n)	
	Quantitative FCIA	Qualitative FCIA	Quantitative FCIA	Qualitative FCIA	Quantitative FCIA	Qualitative FCIA	Quantitative FCIA	Qualitative FCIA	Quantitative FCIA	Qualitative FCIA
1	43.33 (13/30)	26.67 (8/30)	43.33 (13/30)	30.00 (9/30)	53.33 (16/30)	30.00 (9/30)	56.67 (17/30)	50.00 (15/30)	56.67 (17/30)	83.33 (25/30)
2	50.00 (15/30)	50.00 (15/30)	40.00 (12/30)	10.00 (3/30)	50.00 (15/30)	43.33 (13/30)	50.00 (15/30)	33.33 (10/30)	50.00 (15/30)	56.67 (17/30)
3	40.00 (12/30)	56.67 (17/30)	40.00 (12/30)	40.00 (12/30)	46.67 (14/30)	16.67 (5/30)	53.33 (16/30)	16.67 (5/30)	56.67 (17/30)	30.00 (9/30)
CV (%)	9.36	28.94	3.82	46.77	5.44	36.28	5.11	40.82	5.77	38.42

*FCIA* flow cytometric immunobead assay; *CV* coefficient of variation

Direct linear correlations were found between autoantibody concentration and serial dilutions in selected ITP patient samples. The r value for anti-GPIX, GPIb, GPIIIa, GPIIb and P-selectin autoantibodies was 0.98 (p < 0.001), 0.98 (p < 0.001), 0.98 (p < 0.001), 0.98 (p < 0.001) and 0.99 (p < 0.001), respectively (Fig. 2c).

### Platelet autoantibody levels in ITP patients

**Fig. 3:**
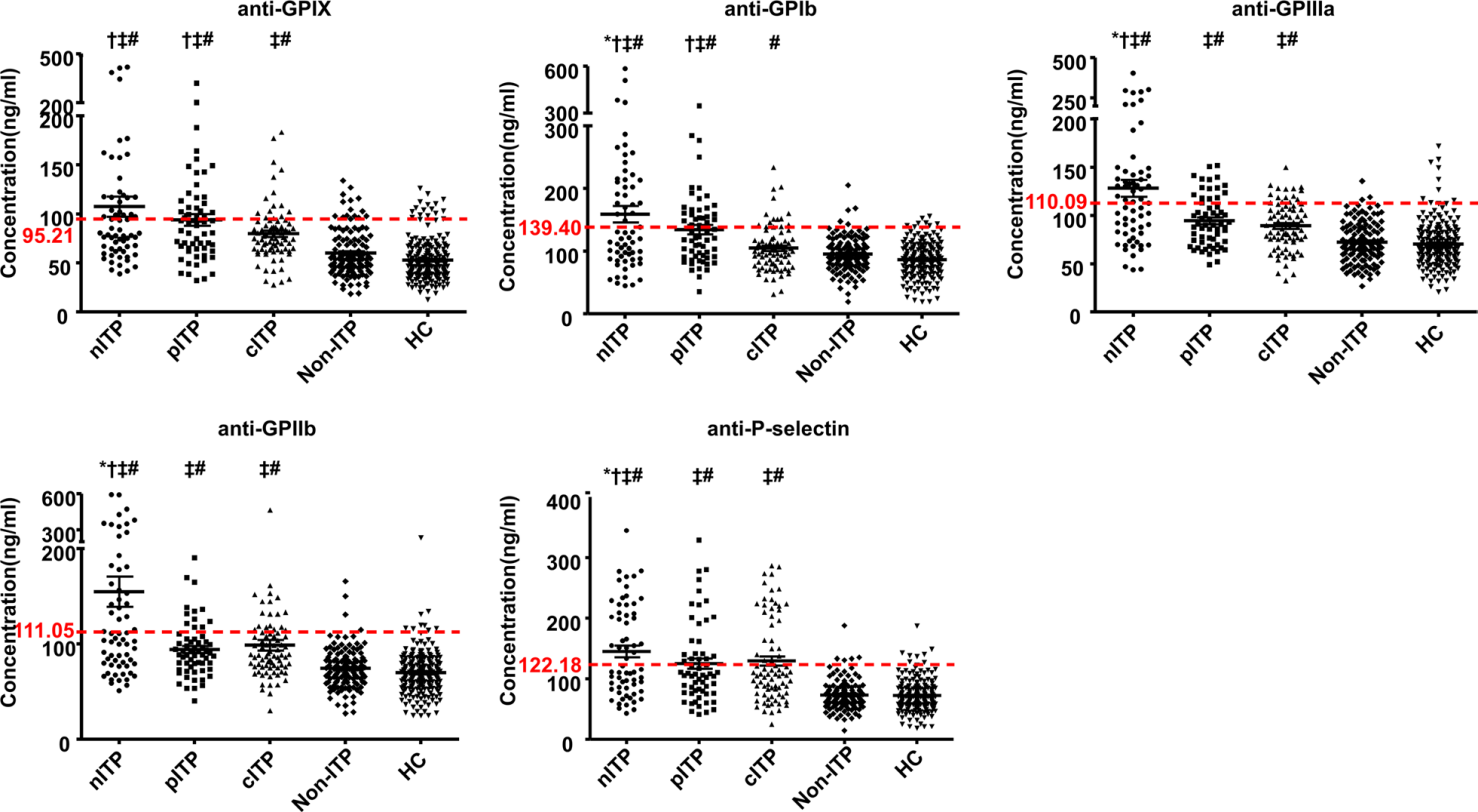
Platelet autoantibodies levels of in ITP patients. Plasma samples from patients with newly-diagnosed (nITP), persistent and chronic ITP (pITP and cITP), non-ITP patients and healthy controls (HC) were assessed by the quantitative FCIA. Increased levels of autoantibodies against platelet GPIX, GPIb, GPIIIa, GPIIb and P-selectin were found in ITP patients compared to non-ITP patients or healthy controls. *p < 0.05 vs. persistent ITP of the same antibody group; ^†^p < 0.05 vs. chronic ITP of the same antibody group; ^‡^p < 0.01 vs. non-ITP of the same antibody group; ^#^p < 0.01 vs. normal controls of the same antibody group. The cut-off values for platelet autoantibodies against platelet GPIX, GPIb, GPIIIa, GPIIb and P-selectin were 95.21, 139.40, 110.1, 111.09 and 122.18 ng/mL, respectively (dashed line)

We used the FCIA assay to measure platelet autoantibodies in a cohort of 201 ITP (newly-diagnosed, persistent and chronic), 126 non-ITP patients and 207 healthy controls. As shown in Fig. 3 and Table 3, increased levels of platelet autoantibodies, including anti-GPIX, GPIb, GPIIIa, GPIIb and P-selectin, were found in ITP patients compared to non-ITP patients and healthy controls (All p < 0.01). The levels of the 5 platelet autoantibodies were all higher in newly-diagnosed ITP patients compared to those in persistent or chronic ITP patients (p < 0.05). Increased levels of anti-GPIX and GPIb autoantibodies were also found in persistent ITP patients compared to those in chronic ITP patients (both p < 0.05), whereas no significant differences were found for anti-GPIIIa, GPIIb and P-selectin autoantibodies (p > 0.05). The autoantibody levels were similar in non-ITP patients and healthy controls (p > 0.05).

**Table 3:** Detection of the different platelet autoantibodies in ITP, non-ITP and healthy controls

Autoantibody category	ITP (n = 201)			Non-ITP (n = 126)	Healthy controls (n = 207)
	Newly-diagnosed (n = 63)	Persistent (n = 60)	Chronic (n = 78)		
Anti-GPIX antibody (ng/mL)	107.47 ± 81.68^†‡§^	93.91 ± 47.44^†‡§^	79.78 ± 56.41^‡§^	59.98 ± 23.46	52.89 ± 21.27
Anti-GPIb antibody (ng/mL)	159.04 ± 104.39*^†‡§^	134.52 ± 57.33^†‡§^	104.84 ± 36.91^§^	95.32 ± 27.44	86.28 ± 26.91
Anti-GPIIIa antibody (ng/mL)	128.24 ± 69.70*^†‡§^	94.60 ± 26.33^‡§^	89.19 ± 24.70^‡§^	72.41 ± 20.93	70.26 ± 23.29
Anti-GPIIb antibody (ng/mL)	154.63 ± 126.02*^†‡§^	94.17 ± 28.82^‡§^	98.54 ± 49.70^‡§^	74.36 ± 21.15	69.83 ± 23.23
Anti-P-selectin antibody (ng/mL)	145.18 ± 73.89*^†‡§^	124.92 ± 66.15^‡§^	129.26 ± 68.73^‡§^	73.29 ± 24.18	72.29 ± 25.31

* p < 0.05 vs. Persistent ITP group with the same antibody

^† ^p < 0.05 vs. Chronic ITP group with the same antibody

^‡ ^p < 0.01 vs. Non-ITP group with the same antibody

^§ ^p < 0.01 vs. Healthy controls group with the same antibody

### Sensitivity and specificity of FCIA assay for ITP diagnosis

**Fig. 4:**
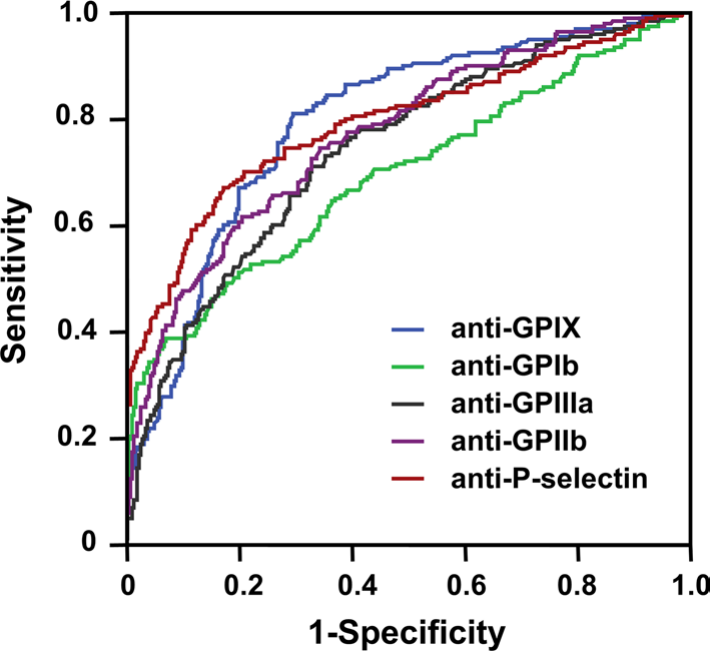
Receiver operating curve (ROC) to assess the specificity and sensitivity of the quantitative FCIA assay for ITP diagnosis. Platelet autoantibodies were analyzed by the quantitative FCIA in 201 ITP patients and 333 non-ITP patients and healthy controls. The AUC values for autoantibodies anti-GPIX, -GPIb, -GPIIIa, -GPIIb and -P-selectin were 0.80, 0.70, 0.76, 0.77 and 0.79, respectively

To evaluate the sensitivity and specificity of the quantitative FCIA assay for ITP diagnosis, ROC curves were analyzed for each type of antibody-coupled microbeads. The cut-off values for platelet autoantibodies against platelet GPIX, GPIb, GPIIIa, GPIIb and P-selectin were 95.21, 139.40, 110.1, 111.09 and 122.18 ng/mL, respectively (Fig. 3). The corresponding values for area under the curve (AUC) were 0.80 (95% CI 0.757–0.825; p < 0.001), 0.70 (95% CI 0.648–0.744; p < 0.001), 0.76 (95% CI 0.720–0.805; p < 0.001), 0.77 (95% CI 0.730–0.813; p < 0.001) and 0.79 (95% CI 0.752-0.835; p < 0.001), respectively (Fig. 4). According to the cut-off values determined by ROC analysis, the positive ratio of antibodies anti-GPIX, anti-GPIb, anti-GPIIIa, anti-GPIIb and anti-P-selectin in our study were 31.84, 32.84, 32.84, 30.35 and 40.30%, respectively, in ITP group, 11.11, 3.97, 3.97, 3.17 and 3.97%, respectively, in non-ITPs and 7.25, 3.38, 5.31, 4.83 and 4.35%, respectively in healthy controls.

### Comparison of quantitative and qualitative FCIA assay

We performed autoantibody detection in the same set of patient samples using a qualitative FCIA assay according to previous description [17]. We compared the specificity, sensitivity and accuracy of these two methods (Table 4). The results were comparable for the sensitivity, specificity and accuracy between the quantitative and qualitative FCIA assays in ITP diagnosis (Table 4).

**Table 4:** Sensitivity, specificity and accuracy based on quantitative and qualitative FCIA assay

Autoantibody category	Case (n)	Sensitivity (%)		Specificity (%)		Accuracy (%)	
		Quantitative FCIA	Qualitative FCIA	Quantitative FCIA	Qualitative FCIA	Quantitative FCIA	Qualitative FCIA
Anti-GPIX (%, n/n)	534	31.84 (64/201)	31.34 (63/201)	91.29 (304/333)	91.29 (304/333)	68.91 (368/534)	68.73 (367/534)
Anti-GPIb (%, n/n)	534	32.84 (66/201)	32.34 (65/201)	96.40 (321/333)	96.40 (321/333)	72.47 (387/534)	72.28 (386/534)
Anti-GPIIIa (%, n/n)	534	32.84 (66/201)	27.86 (56/201)	95.20 (317/333)	97.30 (324/333)	71.72(383/534)	71.16 (380/534)
Anti-GPIIb (%, n/n)	534	30.35 (61/201)	25.87 (52/201)	95.80 (319/333)	97.30 (324/333)	71.16 (380/534)	70.41 (376/534)
Anti-P-selectin (%, n/n)	534	40.30 (81/201)	39.80 (80/201)	95.80 (319/333)	96.10 (320/333)	74.91 (400/534)	74.91 (400/534)
5 antibody combined (%, n/n)	534	73.13 (147/201)	73.13 (147/201)	81.98 (273/333)	82.88 (276/333)	78.65 (420/534)	79.21 (423/534)

*FCIA* flow cytometric immunobead assay

### Comparison of quantitative FCIA and MAIPA assay

Until now the ELISA-based MAIPA assay is the most well-established and specific method for platelet autoantibody detection [25], thus we performed autoantibody detection in a new set of patient samples referred as test set 2, including 41 ITP patients, 37 non-ITP patients and 36 healthy controls (Table 1) using our quantitative FCIA and MAIPA methods. The specificity, sensitivity and accuracy of these two methods were assessed and showed in Table 5. The quantitative FCIA method showed higher sensitivity when using single anti-P-selectin antibody (43.90% vs. 21.95%) or 5 antibodies combined (68.29% vs. 41.46%) than MAIPA assay (p < 0.05) (Table 5), whereas the specificity and accuracy of quantitative FCIA and MAIPA methods was similar (p > 0.05) (Table 5).

**Table 5:** Sensitivity, specificity and accuracy based on quantitative FCIA and MAIPA assay

Autoantibody category	Case (n)	Sensitivity (%)		Specificity (%)		Accuracy (%)	
		Quantitative FCIA	MAIPA	Quantitative FCIA	MAIPA	Quantitative FCIA	MAIPA
Anti-GPIX (%, n/n)	114	43.90 (18/41)^†^	24.39 (10/41)	97.26 (71/73)	97.26 (71/73)	78.07 (89/114)	71.05 (81/114)
Anti-GPIb (%, n/n)	114	39.02 (16/41)^†^	19.51 (8/41)	93.15 (68/73)	94.52 (69/73)	73.68 (84/114)	67.54 (77/114)
Anti-GPIIIa (%, n/n)	114	46.34 (19/41)^†^	26.83 (11/41)	97.26 (71/73)	91.78 (67/73)	78.95 (90/114)	68.42 (78/114)
Anti-GPIIb (%, n/n)	114	51.22 (21/41)^†^	31.71 (13/41)	95.89 (70/73)	94.52 (69/73)	78.82 (91/114)	71.93 (82/114)
Anti-P-selectin (%, n/n)	114	43.90 (18/41)*	21.95 (9/41)	98.63 (72/73)	93.15 (68/73)	78.95 (90/114)	67.54 (77/114)
5 antibody combined (%, n/n)	114	68.29 (28/41)*	41.46 (17/41)	84.93 (62/73)	90.41 (66/73)	78.95 (90/114)	72.81 (83/114)

*FCIA* flow cytometric immunobead assay, *MAIPA* monoclonal antibody immobilization of platelet antigen assay

* p < 0.05; ^†^ p < 0.1 quantitative FCIA vs. MAIPA of the same group

### Platelet autoantibody levels in ITP patients after treatment

**Fig. 5:**
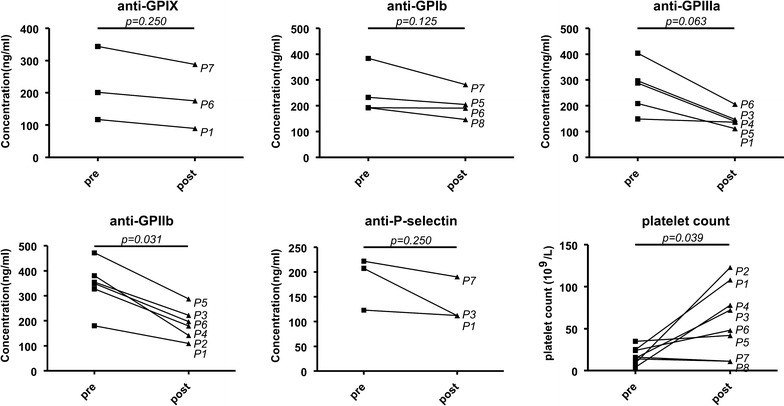
Platelet autoantibodies measured by quantitative FCIA in newly-diagnosed ITP patients at pre- and post- treatment. Platelet autoantibodies were analyzed by the quantitative FCIA in a pilot cohort of 8 ITP patients before (pre) and after corticosteroid treatment (post). P1-P8, ITP patients number. All p values were provided in the figure, and p < 0.05 indicates a significance

The quantitative FCIA assay allowed us to monitor platelet autoantibody levels in a pilot cohort of 8 ITP patients before and after corticosteroid treatment (Fig. 5 and Additional file 1: Table S1). Among the platelet autoantibodies examined, anti-GPIIb autoantibody was the most frequently detected in ITP patients (6 out of 8 patients). The frequencies for anti-GPIIIa, GPIb, GPIX and P-selectin autoantibodies were 5/8, 4/8, 3/8 and 2/8, respectively. In these patients, decreased levels of platelet autoantibodies were observed after the treatment, in which the level of anti-GPIIb antibody was significantly increased (p < 0.05) (Fig. 5). In most of the cases (patients #1–6), the reduced levels of the platelet autoantibodies were consistent with increased platelet counts (p < 0.05) (Fig. 5). Besides, positive for GPIX and GPIb autoantibodies but negative for GPIIb and GPIIIa autoantibodies were found in patients (#7 and #8), however, reduced autoantibody levels were not associated with increased platelet counts (Fig. 5 and Additional file 1:Table S1).

## Discussion

ITP is a common hematological disorder. The etiology of the disease remains unclear. Autoantibodies against platelet membrane glycoproteins play an important role in causing platelet dysfunction and destruction that lead to petechiae, ecchymosis and bleeding in ITP patients [26, 27]. Platelet autoantibody assessment is important not only for distinguishing ITP from non-ITP patients with thrombocytopenia, but also for monitoring ITP patient responses to immune modulation treatment [28]. In addition, according to several most recent studies, the type and persistence of platelet autoantibodies are associated with refractoriness to rituximab or disease severity [6–8]. Thus, the detection of platelet autoantibodies may be helpful in ITP prognosis or personalized treatment, and the further studies about platelet autoantibodies detection in ITP are worthwhile.

To date, several methods have been developed to detect and characterize platelet autoantibodies in ITP patients, which include solid phase red cell adherence (SPRCA) assay [29], platelet immunofluorescence assay [30], antigen-capture assays such as the monoclonal antibody-specific immobilization of platelet antigen assay (MAIPA) [11], and flow cytometric assay [31]. Amony them, the MAIPA assay is the most sensitive and widely used specific platelet antibody detection method [25]. However, due to lacking of quantitative property of this assay could result in high variations within each detection [16, 17]. To circumvent this problem, we developed a novel FCIA assay that uses multiple anti-platelet glycoprotein antibody-coupled microbeads and external human IgG standards to allow quantitative measurement of platelet autoantibodies in plasma samples from ITP patients. Compared to the previous qualitative FCIA assay, the new assay had a better reproducibility, as indicated by CV values ranging from 3.8 to 9.4%, lower than the CV values ranging from 29.9 to 46.8% in the qualitative FCIA assay [17]. Moreover, in the comparison studies, the quantitative and the qualitative FCIA assays had similar sensitivity (73.1 vs. 73.1%), specificity (81.9 vs. 82.9%) and accuracy (78.7 vs. 79.2%) in ITP diagnosis.

Since the ELISA-based MAIPA assay is the most widely used and specific platelet antibody detection method [25], we further performed autoantibody detection in a new set of patient samples using the quantitative FCIA and MAIPA methods. The results showed that our quantitative FCIA had higher sensitivity when using single anti-P-selectin antibody (43.90% vs. 21.95%) or 5 antibodies combined (68.29% vs. 41.46%) and comparable specificity (84.93% vs. 90.41%) and accuracy (78.95% vs. 72.81%) for ITP diagnosis (Table 5). Moreover, according to the cut-off values determined by ROC analysis, the positive ratio when using anti-GPIb/IX and anti-GPIIb/IIIa combination were 62.19% (125/201) in ITP patients and 18.25% (23/126) in non-ITPs, which were higher than 25–39% in ITP patients and 4–9% in non-ITPs detected by MAIPA assay when using anti-GPIb/IX and anti-GPIIb/IIIa combination [9, 32]. These results are consistent with previous studies [14, 15], indicating that the FCIA-based assays have better sensitivity and accuracy than MAIPA and RIA assays in predicting ITP [9, 10, 13, 32].

The most significant improvement of this novel FCIA assay is the ability to quantify platelet autoantibodies in ITP patient plasma. We compared the levels of platelet autoantibodies in different subgroups of ITP disease. It was showed that the concentration of platelet antibodies was lower in persistent/chronic ITP compared to newly diagnosed ITP. As medical treatment history was confirmed in most of persistent and chronic patients (> 90%) (Table 1), it would be expected that historical treatment may account for the lower levels of platelet autoantibodies in patients with persistent/chronic ITP as reported before [19, 33, 34].

Changes in platelet autoantibody levels provide important information in assessing patient responses to treatments and disease prognosis. In a small pilot study with 8 ITP patients, we found that 6 of them had reduced levels of autoantibodies against platelet GPIIb and/or GPIIIa after the corticosteroid treatment. The reduced levels of platelet autoantibodies were associated with increased platelet counts in these patients. In two patients (Fig. 5, patients #7 and #8), platelet counts did not increase after the corticosteroid treatment. In these patients, no GPIIb and GPIIIa autoantibodies were detected, although reduced levels of GPIX, GPIb or P-selectin autoantibodies were observed after the treatment. This result could support the finding that platelet autoantibody specificity may associate with response to conventional treatment, such steroids [34] or intravenous immunoglobulin G [35]. It has been reported that ITP mediated by anti-GPIb/IX is less responsive to treatment [36]. At this time, it is also not clear if there were other disease mechanisms, e.g. altered T cell responses targeting platelets, involved in these two patients.

There are limitations in the present study. First, it should be mentioned that the incomplete separation of platelet glycoprotein complex, e.g. GPIIb/IIIa complex, may result in bias when using a monomer antibody in the setting of our experiment. In order to minimize the bias, optimal detergents for platelet solubilizing would be expected, such as CHAPS [37] used in our preliminary experiment (Additional file 1: Figure S3 and Table S2). However, due to a small sample number, we failed to get a different result with statistical significance and therefore, further investigation should be performed in a large number of samples for verification. Second, case–control study is potentially subject to differential misclassification. Misclassification reduces the ability to detect a difference between the cases and controls. There is also the possibility of bias on the Non-ITP patients because they were presented with different diseases and lack of the other immune related thrombocytopenia, such as systemic lupus erythematosus. In addition, only 8 patients with limited follow-up time were assessed with our quantitative FCIA assay, therefore further studies with more ITP patients and longer follow-up periods are important to assess platelet autoantibody levels in response to different treatments.

## Conclusions

In conclusion, we have developed a novel FCIA assay that allows detecting and quantifying platelet autoantibodies in ITP patient plasma. Monitoring platelet autoantibody level changes should provide important information for assessing the patient response and prognosis in ITP.

## Additional file

**Additional file 1.** Additional figures and tables.

## Abbreviations

ITP: immune thrombocytopenia
FCIA: flow cytometric immunobead assay
FITC: fluorescein isothiocyanate
MFI: mean fluorescence intensity
CV: coefficient of variation
ELISA: enzyme-linked immunosorbent assay
RIA: immunobead-based radioimmune assay
SD: standard deviation
ROC: receiver operating characteristic
AUC: areas under the curves
