# Supplementary material for: Flow cytometric immunobead assay for quantitative detection of platelet autoantibodies in immune thrombocytopenia patients
Source: J Transl Med. 2017 Oct 23;15:214. doi: 10.1186/s12967-017-1317-2 (PMC5654144; doi:10.1186/s12967-017-1317-2)
Supplement: Supplementary file 1 — Additional file 1. Additional figures and tables. [file 12967_2017_1317_MOESM1_ESM.docx]

**Additional Figures**

**Additional Figure S1**

Assessment of the stability of monoclonal antibody-coupled microbeads by flow cytometry. MFI values of the antibody-coated microbeads were assessed by flow cytometry on days 0, 15, 30, 60, 120 and 150, respectively.


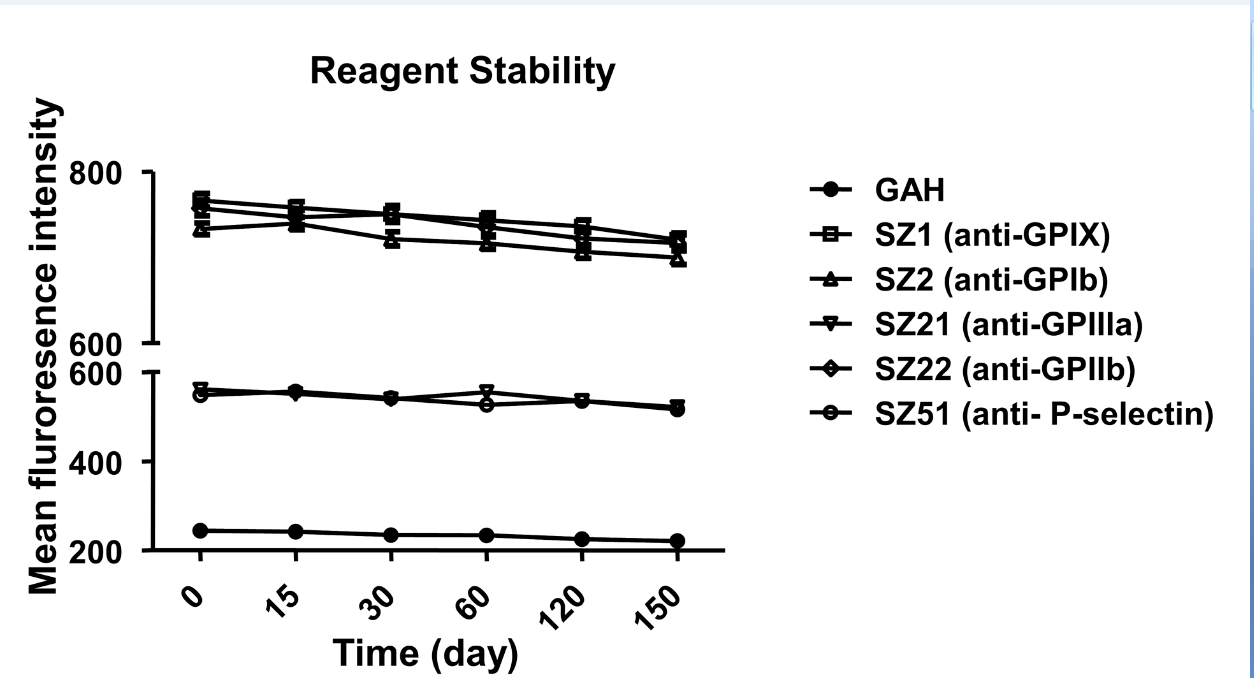


**Additional Figure S2**

Six fitting curves were calculated by using the Log MFI values of six different microbeads of the same diameter but different fluorescence intensities (#1-6) and increasing concentrations of exogenous human IgG. Similar values were found in the equations derived from curve fitting in these results.


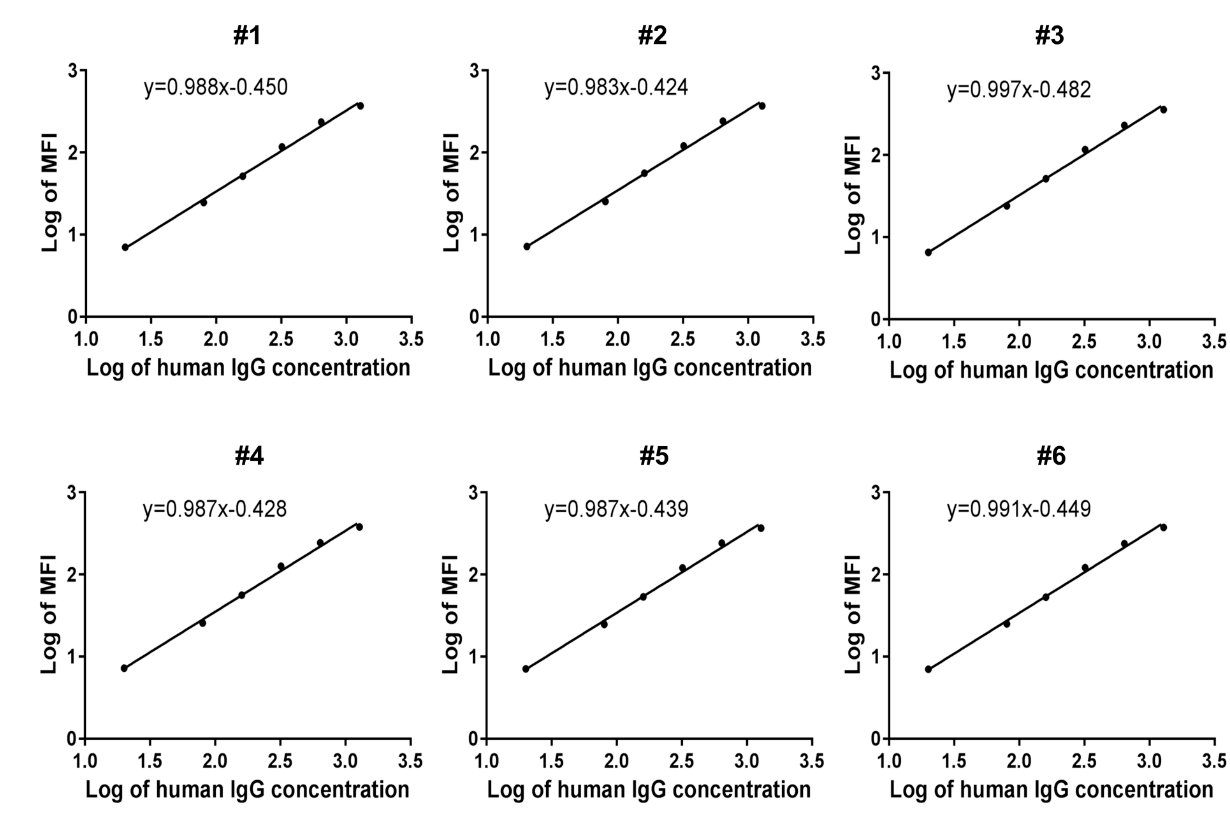


**Additional Figure S3**

Immunoprecipitation of platelet lysates solubilized by different detergents with monoclonal anti-GPIIIa antibody and detected by anti-GPIIb antibody. Precursor of GPIIb (pro-GPIIb) was detected in all the three detergents solubilized lysates (T1, lane 3, 1% Triton X-100; T2, lane 4, 1% Triton X-100+0.1% SDS; CHAPS, lane 5, 1% CHAPS), and mature GPIIb was detected in both Triton X-100 solubilized lysates while it was not detected in CHAPS product. Lane 1, platelet lysate without reducing agent; lane 2, mouse monoclonal IgG control.


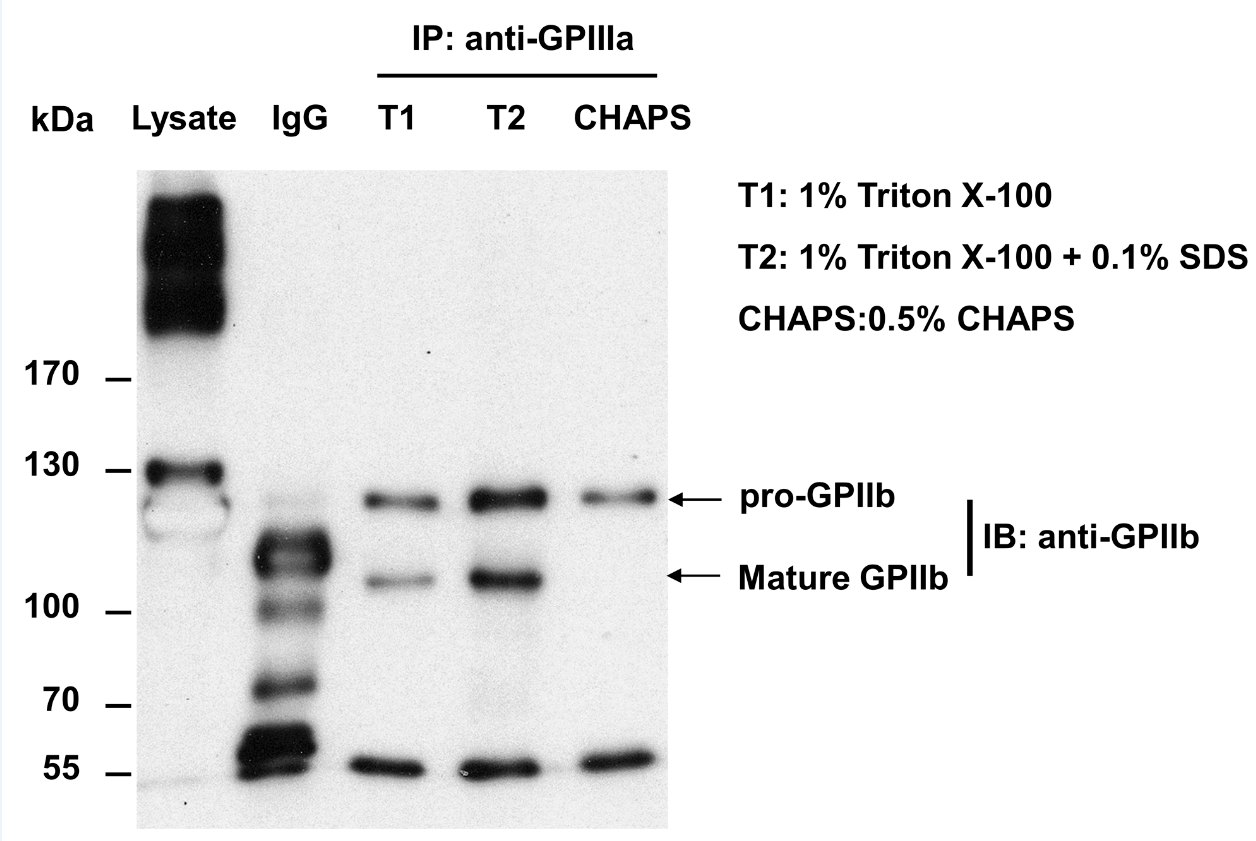


**Additional Figure S4**

Example of comparison of platelet autoantibody anti-GPIIb between disease subtypes of newly-diagnosed (nITP), persistent and chronic ITP (pITP and cITP), non-ITP patients and healthy controls (HC) using “[ “symbols.


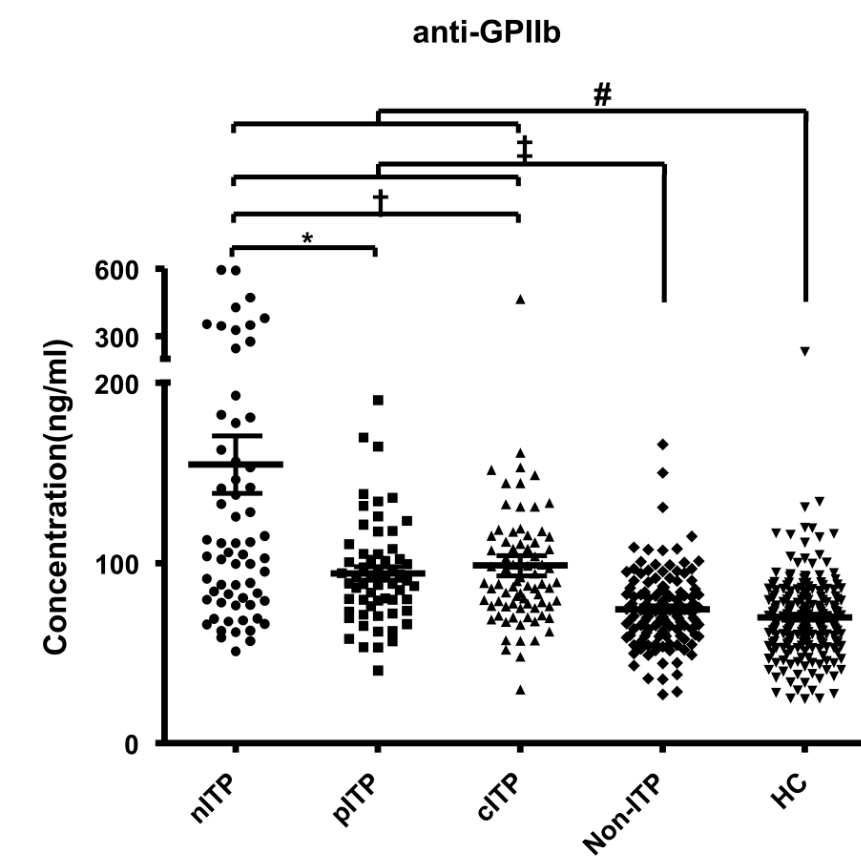


**Additional Table S1. Platelet autoantibodies measured by quantitative FCIA in newly-diagnosed ITP patients at pre- and post- treatment**

| **Patient** | **Age(yrs)** | **Gender** | **Treatment** | **Anti-GPIX antibody (ng/mL)** | | **Anti-GPIb antibody (ng/mL)** | | **Anti-GPIIIa antibody (ng/mL)** | | **Anti-GPIIb antibody (ng/mL)** | | **Anti-P-selectin antibody (ng/mL)** | | **Platelet count (×10^9^/L)** | |
| --- | --- | --- | --- | --- | --- | --- | --- | --- | --- | --- | --- | --- | --- | --- | --- |
|  |  |  |  | Pre | Post | Pre | Post | Pre | Post | Pre | Post | Pre | Post | Pre | Post |
| **1** | 9 | F | steroids | 117.38 | 89.89 | IN | IN | 208.94 | 111.78 | 180.83 | 109.77 | 123 | 112.15 | 25 | 108 |
| **2** | 35 | F | steroids | IN | IN | IN | IN | IN | IN | 380.55 | 141.68 | IN | IN | 9 | 123 |
| **3** | 69 | M | steroids | IN | IN | IN | IN | 296.88 | 147.3 | 354.74 | 222.21 | 207.48 | 111.72 | 15 | 72 |
| **4** | 36 | M | steroids | IN | IN | IN | IN | 288.58 | 140.47 | 327.86 | 179.99 | IN | IN | 4 | 78 |
| **5** | 54 | F | steroids | IN | IN | 233.91 | 204.96 | 148.9 | 136.59 | 471.76 | 287.51 | IN | IN | 35 | 42 |
| **6** | 51 | F | steroids | 201.35 | 175.85 | 192.55 | 190.88 | 404.5 | 206.01 | 348.83 | 196.82 | IN | IN | 24 | 48 |
|  |  |  | steroids, azathioprine |  |  |  |  |  |  |  |  |  |  |  |  |
| **7** | 23 | F | steroids | 344.33 | 288.46 | 384.02 | 282.15 | IN | IN | IN | IN | 221.74 | 190.21 | 16 | 11 |
| **8** | 31 | M | steroids | IN | IN | 192.55 | 147.04 | IN | IN | IN | IN | IN | IN | 14 | 11 |

FCIA: flow cytometric immunobead assay; F: Female; M: Male; IN: invalid values

**Additional Table S2. Comparison of positive rates of platelet anti-GPIIb and anti-GPIIIa by Triton X-100 and CHAPS based FCIA Assays**

|  | **Triton X-100** | | | **CHAPS** | | |
| --- | --- | --- | --- | --- | --- | --- |
|  | GPIIb | GPIIIa | both | GPIIb | GPIIIa | both |
| healthy controls(n=10) | 1 | 1 | 1 | 1 | 1 | 2 |
| ITP patients(n=10) | 5 | 4 | 6 | 7 | 3 | 7 |
